# Methodological approaches in aggregate formation and microscopic analysis to assess pseudoislet morphology and cellular interactions

**DOI:** 10.12688/openreseurope.14894.2

**Published:** 2022-09-20

**Authors:** Fredrik Wieland, Anika Schumacher, Nadia Roumans, Clemens van Blitterswijk, Vanessa LaPointe, Timo Rademakers

**Affiliations:** 1 MERLN Institute for Technology-Inspired Regenerative Medicine, Maastricht University, Maastricht, The Netherlands

**Keywords:** Three-dimensional cell culture, Microwells, Immunofluorescence staining, 3D Software analysis, Cell quantification, ROI quantification

## Abstract

Microscopy has revolutionised our view on biology and has been vital for many discoveries since its invention around 200 years ago. Recent developments in cell biology have led to a strong interest in generating spheroids and organoids that better represent tissue. However, the current challenge faced by many researchers is the culture and analysis of these three-dimensional (3D) cell cultures. With the technological improvements in reconstructing volumetric datasets by optical sections, it is possible to quantify cells, their spatial arrangement, and the protein distribution without destroying the physical organization. We assessed three different microwell culture plates and four analysis tools for 3D imaging data for their applicability for the analysis of 3D cultures. A key advantage of microwell plates is their potential to perform high-throughput experiments in which cell cultures are generated and analysed in one single system. However, it was shown that this potential could be impacted by the material composition and microwell structure. For example, antibody staining was not possible in a hydrogel microwell, and truncated pyramid–structured microwells had increased background fluorescence due to their structure. Regarding analysis tools, four different software, namely CellProfiler, Fiji/ImageJ, Nikon GA3 and Imaris, were compared for their accuracy and applicability in analysing datasets from 3D cultures. The results showed that the open-access software, CellProfiler and Fiji, could quantify nuclei and cells, yet with varying results compared to manual counting, and may require post-processing optimisation. On the other hand, the GA3 and Imaris software packages showed excellent versatility in usage and accuracy in the quantification of nuclei and cells, and could classify cell localisation. Together these results provide critical considerations for microscopic imaging and analysis of 3D cell cultures.

## Plain language summary

High throughput imaging is fast becoming a key instrument for developing therapies for many diseases in the medical research field. A major challenge is the translation from “bench to bedside” for which many scientists rely on high throughput imaging and three-dimensional cell culture techniques to more closely recapitulate human tissue and reduce the need for animal testing. One of the greatest challenges in applying microscopic imaging on larger human tissue mimics, such as 3D cell clusters representing an organ, is the quality of the images and analysis of the acquired information. We show that different three-dimensional cell culture techniques can support high throughput imaging and that not all analysis software solutions are tailored for analysing three-dimensional imaging data.

## Introduction

Microscopy has revolutionised the biological field since its introduction in the late 17th century by Hans and Zacharias Janssen. Only one hundred years later, Antoni van Leeuwenhoek was the first to resolve protists and bacteria. Now, after 200 years of innovation, we can image and reconstruct a whole mouse at high resolution using light-sheet microscopy ^1^ and image living cell cultures in real-time. Light microscopy has become essential in biomedical research, however, the diverse applications require careful selection of the correct microscope for the specimen and the research question.

Microscope technology has, in part, evolved in response to increasingly complex cell culture methods. Initially, *in vitro* research relied on two-dimensional (2D) monolayer cell cultures. Therefore, traditional microscopes were developed to image thicknesses of 10–20 µm. Although tissue explants were studied microscopically beginning in the early 1930s, their assessment relied on cutting, staining and imaging 2D sections. Both 2D cell cultures and explants have major limitations to their use. The 2D cell cultures do not accurately represent many tissues’ morphological and cellular complexity, and many primary cell types lose their phenotype in culture ^1^. Explant studies come with different limitations, such as necrosis after a short time in culture due to their large sizes. To overcome these challenges, scientists have developed three-dimensional (3D) cell culture techniques, and there is significant interest in culturing spheroids and organoids ^2^.

Three-dimensional cell culture methods are applied in a large variety of research fields, such as cancer, development, drug discovery, investigating rare diseases and organogenesis. As a result, a wide range of 3D culture systems have been developed to generate spheroids and organoids. Commonly applied methods are non-adherent microwells ^3, 4^, hanging droplets ^5^, hydrogel/Matrigel embedding ^6, 7^, culture on chip ^8^, bioreactors ^9^, as well as culture on transwells ^10^.

Analytical techniques have evolved concomitantly with 3D culture methods to answer more complex research questions. However, assessing 3D cell cultures is still challenging since most common assays have been optimised for 2D cultures. Morphological assessment and the extraction of spatial information using volumetric microscopy is particularly complex. Tissue processing protocols have to be adapted to avoid the introduction of artefacts, for example, the gradient of chemical fixatives and insufficient antibody penetration that is observed ^11^. The absorbance of light is also significantly different in 3D cultures, resulting in a limited imaging depth that fails to reach the few hundred µm ^12^ to several mm ^10^ needed ^2^. This can be achieved by two-photon microscopy with an immersion objective with a long working distance ^13^, light-sheet microscopy, or the combination of both ^14^. However, since these microscopes are rarely available in standard laboratories, tissue clearing is an alternative to enable further light penetration with single-cell resolution using a standard laser-confocal microscope or a spinning disk confocal microscope ^15, 16^. Overall, tissue processing steps prior to imaging are important for 3D cultures and require extensive optimisation.

Finally, next to the tissue processing, another critical point to consider is that 3D imaging can result in a long imaging time, resulting in large datasets. Consequently, these datasets are more challenging to process and analyse, and require the right software to extract 3D information. Widely applied software for 3D image analysis are open source options, such as CellProfiler ^17^ and ImageJ/Fiji ^18^ or commercial packages such as NIS Elements GA3 ^19^ and Imaris ^20^, with varying degrees of complexity and applicability.

In response to these challenges, this study investigated different microwell-based cell culture options and analysed the resulting data from fluorescence microscopy using the aforementioned software options. We studied a model system of the pancreatic islet, known as a pseudoislet, comprising multiple cell types that self-organize in an aggregate ^21^. This model allows researchers to mimic and study *in vivo*–like cell–cell interactions ^12^ and functionality ^22^ in a context compatible with high-throughput analytical testing to reduce the gap between cell culture, physiological tests and animal research. We used a spinning disk confocal microscope allowing automated, volumetric imaging of pseudoislets. Subsequently, we analysed the number of cells inside the pseudoislet and individual cell localisation in the pseudoislet and cell interactions with collagen IV fibres. The main aim of this study was to evaluate the strength of each software in analysing 3D datasets. In addition, we set out to evaluate commonly requested parameters such as the quantification of nuclei and cells and post-processing and quantification of channel overlay.

## Methods

### Cell culture

Alpha TC1 clone 6 cells were obtained from ATCC (CRL-2934; Manassas, USA). They were cultured in Dulbecco Modified Eagle Medium (6046, Sigma-Aldrich) supplemented with 10% (vol/vol) FBS (Sigma-Aldrich), 2.0% (wt/vol) glucose, 1.5% (wt/vol) sodium bicarbonate, 0.1 mM non-essential amino acids and 15 mM HEPES. INS1E were obtained from AddexBio (San Diego, USA) and cultured in RPMI 1640 (Gibco) supplemented with 5% (vol/vol) FBS, 0.05 mM 2-mercaptoethanol, 10 mM HEPES and one mM sodium pyruvate. Human umbilical vein endothelial cells (HUVECs) were obtained from Lonza (C2519A), cultured in EGM-2 (PromoCell) and used at passage 5. All cell types were cultured in 5% CO _2_ at 37°C and were negative for mycoplasma contamination (BioTool). The generation of stably expressing BFP2-labelled Alpha TC1 cells, and mNeonGreen2-labelled INS1E cells was previously described ^23^.

### Microwell array

Three different microwell array systems were compared: Elplasia 96-well plate (Corning), AggreWell 400 24-well plate (STEMCELL Technologies) and hydrogel microwells 12-well plate (home-made). The Elplasia 96-well plate is a thermoformed polycarbonate microwell plate consisting of 79 microwells per well, and each microwell has a diameter of 500 µm. The AggreWell 400 is a 24-well plate consisting of 1200 microwells per well, and each microwell is 400 µm in diameter. It was prepared with anti-adherence rinsing solution (STEMCELL Technologies). The hydrogel microwells were made using 3% agarose and an elastomeric stamp cast from poly(dimethylsiloxane), as previously described ^4^. Each well consisted of 450 microwells, and each microwell has a diameter of 400 µm.

### Pseudoislet formation

Before cell seeding, every well was washed twice with the modified EGM-2 medium comprising EGM-2 medium supplemented with a final concentration of 10 mM HEPES, 1 mM sodium pyruvate, 0.1 mM non-essential amino acids, 2% (wt/vol) glucose, and 0.05 mM 2-mercaptoethanol. To form pseudoislets, which are three-dimensional aggregates of Alpha TC1 clone 6 cells (BFP2, blue), INS1E (mNeonGreen2, green), and HUVEC cells (unlabelled), the total cell number seeded per microwell was 1500 cells at a ratio of Alpha TC1:INS1E:HUVEC of 1:9:5 ^12, 23^. Cell number was determined by trypan blue exclusion on an automated cell counter (TC20, Bio-Rad Laboratories). All cell types were seeded simultaneously into the microwell arrays, and the plate was centrifuged at 200 × *g* for 4 min to evenly distribute the seeded cells into the microwells. The pseudoislets were cultured in the modified EGM-2 medium for five days with daily medium changes.

### Assessment of immunofluorescence staining inside the microwell arrays

After five days of culture, pseudoislets were fixed in 4% (wt/vol) formaldehyde diluted in PBS for 30 min at room temperature (RT), followed by two washes with PBS. Next, the pseudoislets were permeabilised with 3% (vol/vol) Triton X-100 at RT for 60 min, washed twice in washing solution containing 1% (wt/vol) BSA and 1% (vol/vol) Tween 20 diluted in PBS, and blocked in 5% (vol/vol) goat serum diluted in PBS at RT for 60 min. Finally, the dyes ( Table 1) were diluted in the washing buffer and sequentially stained at 4°C for 24 h. Once the staining was completed, the pseudoislets were washed twice and mounted in ProLong Gold antifade mounting medium (Invitrogen), except for the pseudoislets in hydrogel microwells, which were maintained in PBS for imaging.

**Table 1.** All primary and secondary antibodies and dyes.

Antigen	Host species	Dilution	Source	RRID
Primary Antibodies				
Collagen 4α5	Rabbit (polyclonal)	1/100 (4 µg/mL)	Abcam	Abcam (ab231957)
Secondary Antibodies				
Rabbit IgG Alexa Fluor 647 nm	Goat (polyclonal)	1/500 (4 µg/mL)	Invitrogen	Invitrogen (A-21245)
Dyes				
DAPI	-	0.7 µg/mL	Sigma-Aldrich	Sigma-Aldrich (32670-5MG-F)
Sytox Orange Nuclei Acid Stain	-	1/1000 (5 µM)	Invitrogen	Thermo Fisher (S11368)
Alexa Fluor 647 Phalloidin	-	1/200 (0.3 µM)	Invitrogen	Thermo Fisher (A22287)

### Preparation of fluorescently stained pseudoislets for analysis

The second method used to stain pseudoislets involved careful collection of the fixed pseudoislets with a 1 mL pipette out of the microwell array into 1.5 mL microcentrifuge tubes following two washes with PBS. Prior to the collection, the tip was rinsed in a solution of 3% FBS diluted in PBS to prevent the attachment of pseudoislets. Inside the microcentrifuge tubes, the pseudoislets were treated as described above in terms of permeabilization and blocking. Then, the primary and secondary antibodies and dyes ( Table 1) were diluted in the washing buffer and sequentially stained at 4°C for 24 h with primary antibodies and 48 h with secondary antibodies. The pseudoislets were washed three times with washing buffer before the secondary antibody. Once the staining was completed, the pseudoislets were carefully collected with a 1 mL pipette tip, transferred into CELLview dishes (Greiner Bio-One) and mounted in ProLong Gold antifade mounting medium.

### Microscopy

Optical sections (z-stacks) of the pseudoislets were obtained on a Nikon Eclipse Ti-E inverted microscope equipped with a 40×/1.3 NA immersive oil objective (Plan Fluor 40× Oil DIC H N2, Nikon Instruments) and spinning disc X-Light2 (CrestOptics). The light source was an LED-based Spectra (Lumencor), and images were captured using a Photometrics Prime 95B CCD camera. BFP2 was detected with ExW: 395 nm and EmW: 454 nm and exposure of 200 ms and camera setting set to binning of 2×2 with the filter cube DAPI. mNeongreen2 was detected with ExW: 480 nm and EmW: 517 nm and exposure of 50 ms and camera setting set to binning of 2×2 with the filter cube FITC. Sytox orange nuclei acid stain was detected with ExW: 568 nm and EmW: 570 nm and exposure of 20 ms and camera setting set to binning of 2×2 with the filter cube TRITC. Collagen IV was detected with ExW: 647 nm and EmW: 665 nm and exposure of 70 ms. The camera setting was set to binning of 2×2 with the filter cube Cy5 HYQ. Shading correction was performed per channel prior to image acquisition, and the camera sensor was cropped using an ROI to remove the spinning disk edges from the field of view. For z-stacking, z-step size was set to 0.3 µm distance; z-stack ranged from 0–90 µm in the depth of the pseudoislets.

### Microscopy analysis

Cell quantification was completed on digital images using four different software packages: Fiji ^24^, CellProfiler ^25^ (Broad Institute, Cambridge, MA, USA), NIS Elements GA3 (Nikon Instruments), Imaris 9.5.0 (Bitplane, South Windsor, CT, USA). Manual quantification by eye was used a reference. Before analysis, the digital images were post-processed with background noise reduction by individual software. Subsequently, each cell type was isolated by thresholding the fluorescence channel to generate binary masks. Finally, different masking algorithms were applied depending on the software to quantify the cell number, distribution of cells in the core and mantel of the pseudoislet, and cell–matrix interactions.

***Manual quantification.*** Images were opened in Fiji and the cell counter plugin (2D) was used to mark cells in the datasets manually by scrolling through the z-stack. Then, at every 4.5 µm step, quantified cells were scrutinized for double counts. To quantify core and mantle, we distinguished the mantle by assessing cell localisation depending on whether they were one cell layer away or included in the peripheral cell layer of the pseudoislet (resulting in ~80% core, ~20% mantle).

***Fiji.*** Images were first separated into single channels and post-processed with subtracting background, set to 50 pixels. Next, the quantification of nuclei and cells was done by using the plugin 3D object counter. Finally, the threshold was set individually for each channel and dataset with a filter size >250.

***CellProfiler.*** Images were analysed using CellProfiler 4.1.3 with a custom-made pipeline. Intensity values of each image were rescaled to the full intensity range (from 0 to 1) to improve downstream analyses. In addition, a median filter with filter size 5 was added to reduce background noise and homogenise the signal within the nucleus. Nucleus morphology was captured by the adaptive two-class Otsu thresholding method, and nuclei quantification was done using two different modules: one for measuring nucleus signal intensity and the other for measuring the nucleus size and shape.

***NIS Elements GA3.*** Images were analysed using NIS Elements AR 5.20.1 with a custom-made pipeline. For nuclei and cell count, the relevant channels were processed using a rolling ball set to 27 µm, intensity in z was equalised, and a Gaussian LaPlace filter (power: 2.0) was applied. The signal was then thresholded for both channels, and total nuclei and INS1E cells were counted. Centroids for nuclei and INS1E cells were established in this same pipeline to determine core or mantle localisation. Core/mantle was determined by performing detection of the whole spheroid based on thresholding, after which an erode function was used to create a core/mantle boundary, which was automatically set by the user to a volume distribution of 60% core volume versus 40% mantle volume. For cell–ECM interactions, the INS1E channel was processed using a rolling ball set to 27 µm, intensity in z was equalised, and a Gaussian LaPlace filter (power: 2.0) was applied, after which it was thresholded for detection of the cells. The collagen IV channel was smoothened using ‘fast smooth’ and subsequently thresholded. Using the ‘aggregate children’ function, interactions between ECM (parent) and cells (children) was quantified based on ‘children interacting with parent’.

***Imaris.*** The image quantification in Imaris 9.5 followed the provided wizard to identify nuclei using spot function and surface function to identify cells. Each image was post-processed with background-noise reduction set to 60 µm for nuclei and cells. A normalisation step was performed for each channel to adjust the signal to noise ratio between the z-stack signal in the pseudoislets. The estimated xy diameter of the nuclei was set to 6 µm within the ‘spot’ function, and additional background subtraction was included in the quantification. The thresholds were set individually for each image, and the filter set was used to identify individual nuclei in the pseudoislet. The ‘surface’ function and detail smoothness were set to 0.6 µm, and background subtraction with a 16 µm diameter for analysed spheres. Thresholds were individually set with a split touching object of a diameter of 8 µm. The quality filter was adjusted to count all thresholded areas and the size filter filtered out any object size below 353 µm ^2^.

In addition, quantification of the ECM protein collagen IV was carried out to assess cell–ECM interaction. The collagen IV signal was post-processed with background noise reduction set to 20 µm. After identifying nuclei, cells and collagen IV, the function ‘object-object statistic’ was used to identify overlapping objects distance toward each other. A maximum distance of 0 µm was applied to quantify the cell–matrix interaction between the cells and collagen IV. In order to identify the localisation of the cells, the pseudoislet volume surface was divided into core and mantle. First, it was necessary to erode the nuclei channel to a threshold, in which the total surface of the pseudoislet was identified. Then, the channel intensity filter was set to automatically divide the calculated volumetric surface into 80% core and 20% mantle to distinguish the regions. Then the function object-object statistic identified in which region each cell was located.

### Statistical analysis

Data were reported as mean or 10 ^th^–90 ^th^ of mean. Statistical analysis was carried out in Prism software (version 8.1; GraphPad, La Jolla, CA, USA), with a post-hoc test of an ANOVA, determined by an unpaired Dunnett and Tukey t-test when *P* < 0.05. The free-to-use statistics program JASP could be used for these analyses.

## Results

### Selection of a suitable microwell array

**Figure 1.**
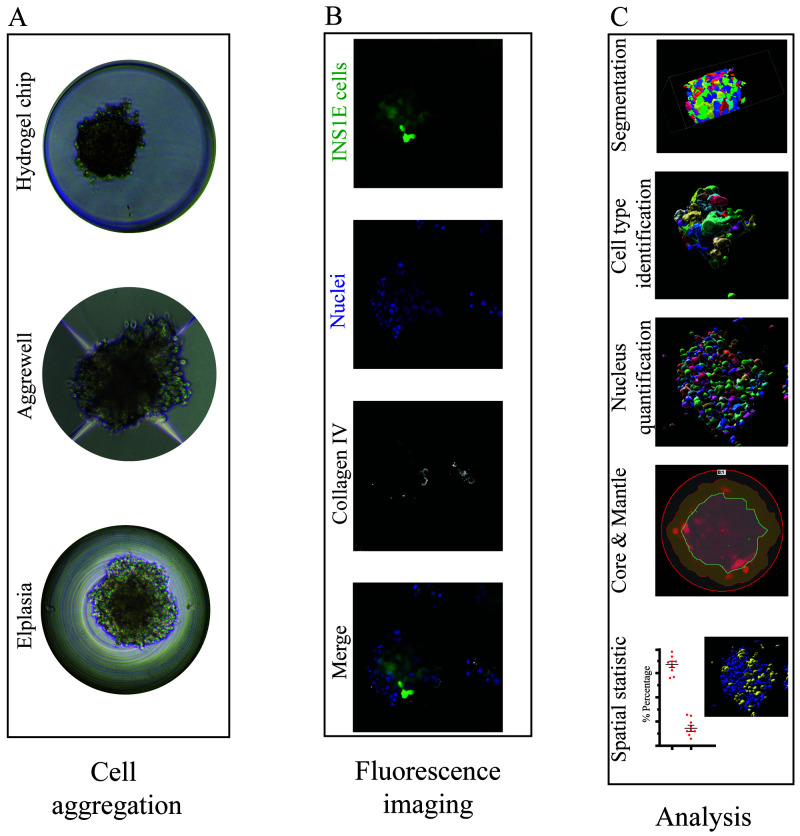
Overview of microwell culture platforms and analysis by microscopy. ( **A**) Three different microwell systems were compared for their support of the formation of aggregates and immunofluorescence staining. ( **B**) The different cells were quantified by max intensity projection of 70 z-stack images (z-distance of 0.3 µm), and each image displayed the aggregated INS1E cells (mNeonGreen2, green), nuclei (Sytox orange, blue) and collagen IV (white). ( **C**) Illustration of the software workflow beginning with cell masking followed by segmentation and quantification of the nuclei and cell types. Subsequently, the regions of core and mantle and overlapping signals were analysed.

**Figure 2.**
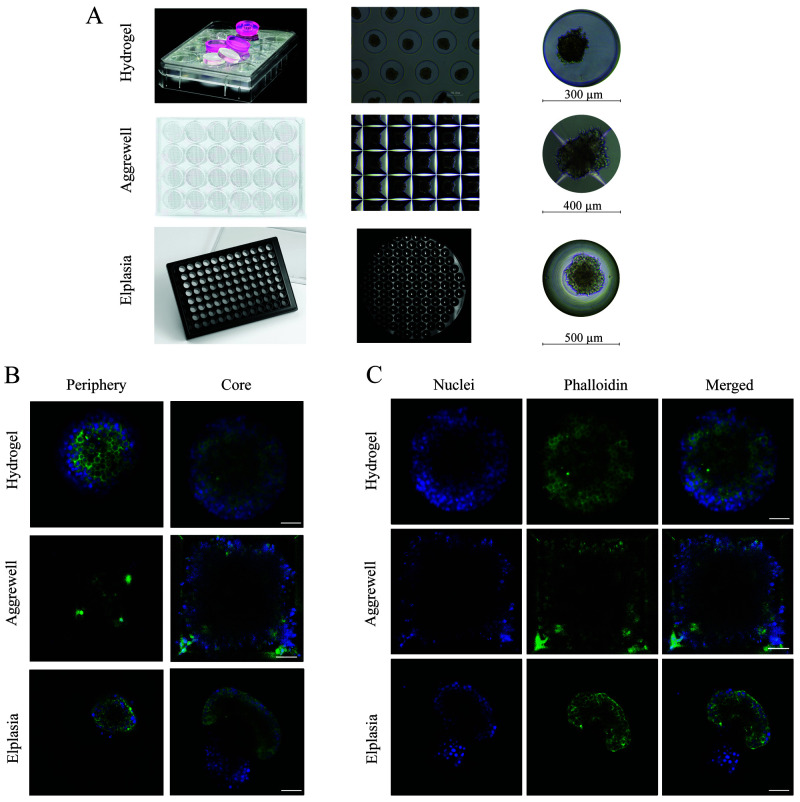
Comparison between the three different microwell systems. ( **A**) Overview of the plate layout of each culture system and the individual microwell size. The hydrogel microwell is a 24-well plate agarose-based microwell system with 450 microwells per well, and each microwell has a diameter and height of 300 µm and 500 µm, respectively. The AggreWell is a 24-well plate truncated pyramid microwell structure with 1200 microwells per well, and every microwell has a diameter of 400 µm. The Elplasia is thermoformed microwells in a 96-well plate with 79 microwells per well, and each microwell has a diameter of 500 µm and a height of 400 µm. ( **B**) Comparison in imaging depth in each microwell system from the periphery (0–5 µm from the surface) compared to the core (60 µm from the surface), using a 40×/1.3 NA immersive oil objective. ( **C**) The images demonstrate the result after F-actin (phalloidin, green) and nuclei (DAPI, blue) staining inside the microwells, where each well was washed three times before imaging.

We were interested in generating pseudoislets, cell aggregates of three different cell types that resemble an islet of Langerhans. In order to do this, using non-adhering microwells to support optimal cell self-aggregation is crucial. Therefore, three different technologies: a thermoformed polycarbonate (Elplasia), a truncated pyramid microwell structure (AggreWell 400) and a hydrogel microwell (agarose-formed microwells made in-house), were compared for their optical properties and permissibility to cell staining ( Figure 1A, B). All three systems were able to generate pseudoislets ( Figure 2A). The hydrogel microwell enhanced cell aggregation, likely because the cells could not adhere to the polysaccharides ( Figure 2A).

When assessing their performance in fluorescence imaging for microscopy, all three culture systems allowed staining with DAPI (nucleus) and phalloidin (F-actin). However, thorough washing was necessary to avoid background signal; in the Aggrewell 400, some background staining remained on the edges of the microwells ( Figure 2B). Antibody staining was only compatible with the polymer-based Aggrewell and Elplasia microwells. Since the antibodies were adsorbed to the agarose in the hydrogel microwell, no successful staining could be achieved (data not shown). For imaging resolution, both the hydrogel and Elplasia microwell arrays showed good single-cell resolution imaging even at a depth of 60 µm into the pseudoislet. The truncated pyramid structure of the Aggrewell caused a low signal-to-noise ratio for identifying single cells with suitable analysis quality ( Figure 2B–C). That resulted in a shortfall in attaining single-cell resolution, which can be seen in the low immunofluorescence signal in the centre of the pseudoislets as less light was reflected back to the camera sensor due to light scatter from the truncated pyramid structure. 

### Analysis of three-dimensional single-cell quantification

High-throughput imaging of microwell-based pseudoislets results in large data sets that require a more automated method of data analysis, e.g., the quantification of nuclei and cells. Thus, we compared four different software packages for their ability to extract 3D information from z-stacked immunofluorescence images: GA3, Imaris, Fiji, and CellProfiler ( Figure 1C, D).

**Figure 3.**
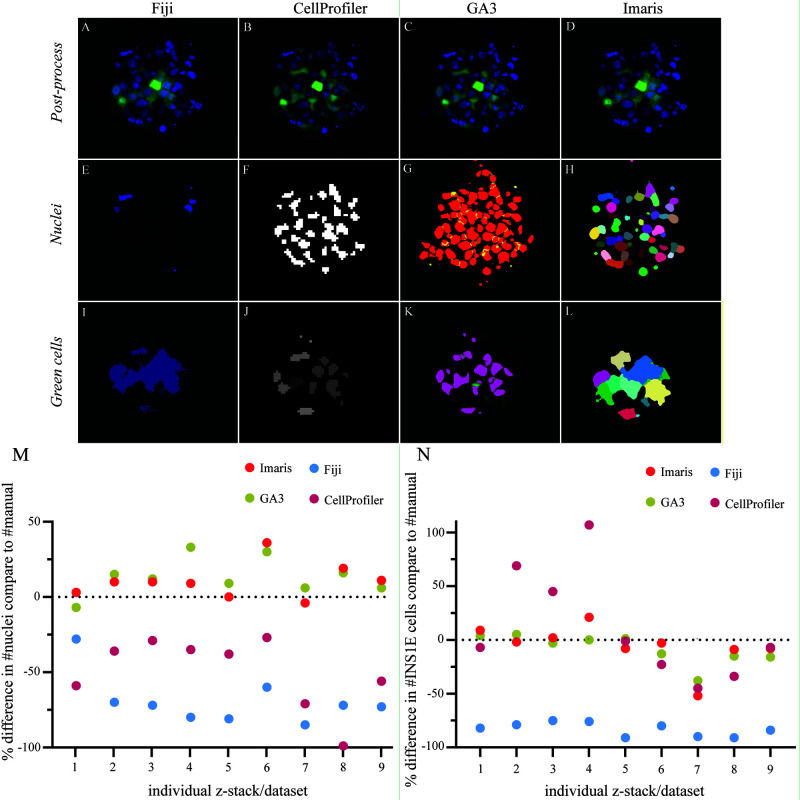
Comparison in the software performance for segmentation and quantification. ( **A**– **D**) Post-processed image of the aggregated INS1E cells (green) and nuclei (blue) with applied background noise reduction and immunofluorescence signal normalisation by each software. ( **E**– **H**) The threshold segmentation of individual nuclei ID represents the thresholding success for all four software, Fiji, CellProfiler, GA3 and Imaris, respectively. ( **I**– **L**) Focus on cell segmentation between single cells in the aggregate and how well each of the four different software could identify single cells. ( **M**) The software was compared to manual counting for their performance to quantify nuclei and correct for multiple counts. The data represent an over– or underestimated count of nuclei in the different images containing aggregates of cells. The software GA3 and Imaris overestimated the count of nuclei with an average of 13% and 10%, respectively. Fiji and CellProfiler underestimated the count of nuclei with an average of 69% and 50%, respectively. ( **N**) A comparison between the software for their success segmenting single cells in the cell clusters. GA3 and Imaris had close quantifications to the manual cell count with a slight underestimation of 8% and 6%, respectively. CellProfiler showed results close to GA3 and Imaris along with larger variation in over– and underestimations of the cell count with an average of 12% overestimation. Fiji showed a consistent underestimation of the cell count with an average value of 83% from the manual count. Results are calculated by the relative change, and the data comprised nine z-stack data sets.

**Figure 4.**
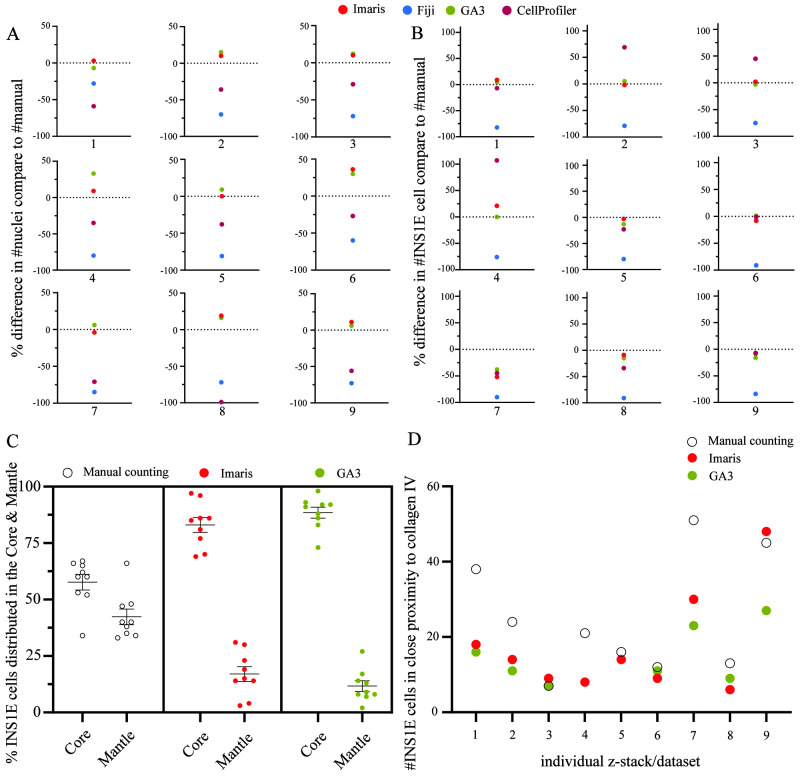
Comparison of the software performance. ( **A**) GA3 and Imaris produced equal quantification of the nuclei except in image four, where GA3 slightly overestimated the count compared to Imaris. In most z-stacks, CellProfiler was closer to the manual count than Fiji except in dataset one and eight. However, overall, they underestimated the number of nuclei. ( **B**) For the quantification of single cells, both GA3 and Imaris, resulted in equal counts as the manual count except in dataset four and seven. However, CellProfiler showed equal accuracy as GA3 and Imaris in many z-stacks, except in dataset two, three, four and eight. Fiji had a consistent underestimation of ~83% in all datasets. ( **C**) Comparing the distinction between core and mantle in the aggregates. The manual counting had a lesser distinction between the core and mantle distribution of the INS1E cells compared to both GA3 and Imaris that used a percentage area distribution mask to quantify the distribution of the cells in each area. This resulted in an 89% and 17% core versus mantle distribution in GA3 and 83% and 17% in Imaris. ( **D**) Another method was to quantify overlapping signals between two immunofluorescence channels within a 0 µm distance. The GA3 and Imaris software resulted in equal count except in datasets nine and seven. However, in most situations, the manual quantification had a higher count than GA3 and Imaris. Only dataset three, five, six and eight had comparable results as the software. Results are calculated by the relative change, and the data set included nine z-stack data sets.

Pseudoislets from all three microwell platforms were transferred to CELLview dishes and mounted with Prolong Gold mounting medium. Each software post-processed the images to remove background noise ( Figure 3A–D). It should be noted that, in our hands, the CellProfiler post-process plugin solution was not effective to improve nucleus or cell detection. Therefore, the GA3 post-processed images were used for the CellProfiler analysis to improve the segmentation of the nuclei and cells. Fiji, GA3 and Imaris applied rolling ball background subtraction to isolate the fluorescence signal from the background noise and enhance the signal-to-noise ratio ( Figure 3A, C, D). In addition, Imaris normalised the fluorescence signal between every z-stack image to compensate for signal loss further into the tissue ( Figure 3D). GA3 applied identical intensity compensation and a Gaussian LaPlace filter to enhance the borders of nuclei and cells ( Figure 3C). Manual thresholding was performed to quantify nuclei and INS1E cells in the pseudoislets ( Figure 3E–H). Quantification of the INS1E cells showed a similar ability of CellProfiler and GA3 to extract the INS1E cells ( Figure 3J, K). Both GA3 and Imaris quantified INS1E very close to manual counting, with underestimations of 8% and 6%, respectively. CellProfiler performed better for INS1E cell detection than nuclei quantification, which had a 12% overestimation in cell detection compared to manual counting. However, the variability was high between the different samples ( Figure 3N, Figure 4B). Imaris and Fiji could less clearly distinguish cell borders, causing an expanded fluorescence area estimation, which resulted in larger masks. However, Imaris achieved better cell separation than Fiji, which resulted in an accurate cell count, as the cell clusters were separated into single cells ( Figure 3I, L). Fiji underestimated the number of INS1E cells in the pseudoislet by 83% compared to the manual count.

For quantification of nuclei, Fiji was less capable of quantifying the nuclei located in the inner core of the pseudoislet ( Figure 3E). CellProfiler and Imaris showed similar masking of nuclei, although Imaris could better separate nuclei in close proximity to each other ( Figure 3F, H). GA3 identified more nuclei and showed good separation between closely localized nuclei ( Figure 3G). Overall, the performance of the four different software packages in quantifying nuclei resulted in two groups. The first group, GA3 and Imaris, overestimated nuclei, with 13% and 10% overestimations, respectively, compared to manual quantification. In the second group, CellProfiler and Fiji both underestimated the number of nuclei in the pseudoislets, with 50% and 69% underestimations, respectively ( Figure 3M, Figure 4A). Overall, Fiji substantially underestimated both INS1E cells and nuclei compared to manual counting.

Next, we evaluated the software packages for their ability to extract spatial information from the cells. More specifically, we were interested in assigning the cells to different regions in the pseudoislet architecture (core or mantle) and their proximity to the extracellular matrix protein, collagen IV. Due to the variability in the results of Fiji and CellProfiler in our previous analyses and the limitation in the possibility of analysing a specific region of interest, these two software packages were excluded. In the execution of dividing the pseudoislet volume into core and mantle regions, they were distinguished as 60% and 40% in GA3 and 80% and 20% in Imaris, respectively. The manual quantification (~80% core, ~20% mantle) diverged from the software, as the defined quantification parameters of core and mantle were divided by assigning the outer two cell layer the mantle (i.e., the periphery) and all remaining cells were assigned to the core. Both GA3 and Imaris showed good performance in quantifying the INS1E cells in the two regions ( Figure 4C). Both GA3 and Imaris showed occasionally identical quantification of INS1E cells, e.g., datasets 4 and 5.

The results of the co-localisation analysis between INS1E cells and collagen IV are summarised in Figure 4D. The analysis with GA3 and Imaris showed an average of 14 and 17 INS1E cells co-localised with collagen IV, respectively, lower than an average of 25 INS1E cells identified by manual counting. Overall, GA3 and Imaris showed mediocre performance in identifying their extracellular matrix interactions. This could be affected by the masking settings in the software, which are created by the intensity threshold. The effect of changes in the threshold can result in larger or smaller masks, in all Z, X and Y axes around the cells and collagen IV, which would result in fewer or more interactions.

Together, these data indicate the complexity of analysing 3D data and generating reliable outcomes. In summary, these results show that software solutions such as GA3 and Imaris can deliver consistent results. However, we would like to emphasise that thresholding is very important for image-based analysis regardless of the software used.

## Discussion

Three-dimensional culture systems have revolutionised *in vitro* research approaches and allow better translation of *in vitro* work into *in vivo* results. This movement sows the seeds for more complexity in cell culture experiments and shortens the timeframe between “bench to bedside.” However, the innovation of using 3D cell culture introduces new challenges, especially for implementation of microscopy, as both the imaging setup and the analysis workflow need to be carefully considered. For our application with pseudoislets, the current manuscript investigated the applicability of the different microwell culture systems in terms of staining and subsequent microscopic imaging. Subsequently, four analysis software packages were compared, focusing on quantifying nuclei and INS1E cells inside the spheroids. In addition, the software was evaluated for its ability to analyse the distribution of cells in different areas or correlate channel-over-channel identification.

We observed a striking performance difference between different software packages when segmenting nuclei and INS1E cells within the pseudoislets. Fiji showed the lowest success rate in quantifying both nuclei and INS1E cells in 3D, which was most likely due to the difficulties in segmentation objects with very similar intensities and close proximity. This resulted in a larger positive area, which is then classified as one object in the quantification. To resolve that issue, we applied the watershed function in Fiji to segment between cells. However, Fiji had limitations in working with 3D data, as multiple cells were quantified twice or even three times each in the z-stack. In this study, we also compared different available plugins for 3D quantification in Fiji and selected the standard “3D object counter” plugin to quantify nuclei and INS1E cells.

CellProfiler underestimated the number of nuclei in every dataset, possibly due to the close proximity between the nuclei that resulted in their quantification as a single nucleus ( Figure 3F). This discrepancy was less prominent for larger objects, such as the INS1E cells, with an average of 12% overestimation compared to manual counting. Again, though, it should be noted that CellProfiler fluctuated between over- and underestimation of the datasets. The Nikon GA3 module and Imaris provided the closest estimation of nuclei and INS1E cell quantification to the manually counted results.

Despite its limitations in our evaluations, Fiji has the potential to produce better results, which has also been shown in other publications ^26– 29^. However, Fiji needs to be adapted to individual user circumstances, and therefore cannot be directly applied by every user. On the other hand, Fiji makes it a good tool for groups with knowledge in programming macros for specific research purposes, as these macros allow automated analysis. To conclude, Fiji is a flexible software solution for many different users. However, it is important not to expect that Fiji would be one solution that fits them all.

CellProfiler is an open-access software package that focuses on quantitative measurements and creates pipelines for fast analysis in an interface that does not require training. These advantages offer great potential for CellProfiler, especially if improvements in its analysis of 3D datasets can be made.

In our study, once the pipeline was established, the analysis was much faster and could be done in less than 1/6 of the time, which applied for CellProfiler, GA3 and Imaris. GA3 and Imaris were more versatile in their current state and allowed us to do other types of analysis, such as cell–matrix interactions and assessing cell counts related to the architecture of the pseudoislets. Analysis of the distribution of the INS1E cells between the two regions, core and mantle, resulted in a better performance with the software than manual quantification ( Figure 4C). In our experience, GA3 had a better functional interface to automatically apply the defined region of core/mantle in every dataset compared to Imaris, which required additional manual input.

The following analysis entailed the identification of the ECM protein collagen IV adjacent to INS1E cells. GA3 and Imaris showed comparable results between the datasets, in which the cells needed to overlap with the collagen IV, i.e. have <0 µm distance to the collagen IV to be quantified in order to accurately determine interaction between cells and ECM. Our manual count counted more INS1E cells, but this quantification was difficult due to the subjectiveness of the distance between the INS1E cell and collagen IV signal ( Figure 4D). To compare the 3D interaction analysis with a manual count setup is challenging, as the scale of <0 µm distance is difficult to measure in a 3D dataset. Consequently, using manual quantification as a relative standard is irrational in this analysis, as the distance of the two signals are relative to the user’s estimation. These results therefore reflect the accuracy, repeatability and versatility these two software provides when working with 3D datasets. However, it is essential to note that even though these two software solutions provide a user-friendly interface, they still require user experience in creating and setting up analysis protocols.

In summary, the current data highlight the importance of thoughtful selection when deciding on 3D culture and analysis setup, as not every setup can deliver the same output. In our microwell systems for our pseudoislet culture, we experienced the best balance between staining and imaging quality when using the Elplasia microwell plates, though these were the most difficult microwells from which to collect the pseudoislets. The assessment of the four different software packages has extended our knowledge about the performance but also limitations when using software solutions to analyse 3D datasets. The insights gained from the comparisons between the software may assist other researchers, as, in our hands, GA3 and Imaris outperformed Fiji and CellProfiler, but are not as easily accessible. When designing a research question, we suggest that clearly defining the required analysis and executing some testing could be valuable, as software such as Fiji and CellProfiler can still be well adapted for 3D cell quantification with necessary extensive optimisation. Nonetheless, GA3 and Imaris provided faster solutions with a user-friendly interface without training, and after a short learning curve, they provided a post-process solution that improves the segmentation of cells.

## Data Availability

DataverseNL: Methodological approaches in aggregate formation and microscopic analysis to assess pseudoislet morphology and cellular interactions, https://doi.org/10.34894/N1EATZ. Data are available under the terms of the Creative Commons Zero "No rights reserved" data waiver (CC0 1.0 Public domain dedication).
