# Supplementary material for: Methodological approaches in aggregate formation and microscopic analysis to assess pseudoislet morphology and cellular interactions
Source: Open Res Eur. 2022 Sep 20;2:87. Originally published 2022 Jul 13. [Version 2] doi: 10.12688/openreseurope.14894.2 (PMC10446072; doi:10.12688/openreseurope.14894.2)
Supplement: Peer review history

# Peer review: Reviewer response for version 2

**DOI:** 10.21956/openreseurope.16350.r30123

**Published:** 2022-09-29

**Authors:** Jose J. Muñoz

**Affiliations:** 1 Laboratori de Càlcul Numèric (LaCàN), Department of Mathematics, Polytechnic University of Catalonia, Barcelona, Spain

The authors have responded to my comments and doubts, which have been clarified.

Is the rationale for developing the new method (or application) clearly explained?

Yes

Is the description of the method technically sound?

Partly

Are the conclusions about the method and its performance adequately supported by the findings presented in the article?

Yes

If any results are presented, are all the source data underlying the results available to ensure full reproducibility?

Partly

Are sufficient details provided to allow replication of the method development and its use by others?

Yes

Reviewer Expertise:

Biomechanics, Cell rheology, Tissue mechanics, Numerical Analysis, Optimisation.

I confirm that I have read this submission and believe that I have an appropriate level of expertise to confirm that it is of an acceptable scientific standard.

# Peer review: Reviewer response for version 2

**DOI:** 10.21956/openreseurope.16350.r30124

**Published:** 2022-09-22

**Authors:** Janne T. Koivisto

**Affiliations:** 1 Division of Pathology, Department of Laboratory Medicine, Karolinska Institutet, Stockholm, Sweden

Approved, no further comments.

Is the rationale for developing the new method (or application) clearly explained?

Yes

Is the description of the method technically sound?

Yes

Are the conclusions about the method and its performance adequately supported by the findings presented in the article?

Yes

If any results are presented, are all the source data underlying the results available to ensure full reproducibility?

Yes

Are sufficient details provided to allow replication of the method development and its use by others?

Yes

Reviewer Expertise:

Expertise in developing 3D cell culture systems (especially hydrogels), human cell culturing, and 3D imaging

I confirm that I have read this submission and believe that I have an appropriate level of expertise to confirm that it is of an acceptable scientific standard.

# Peer review: Reviewer response for version 1

**DOI:** 10.21956/openreseurope.16096.r29791

**Published:** 2022-09-06

**Authors:** Jose J. Muñoz

**Affiliations:** 1 Laboratori de Càlcul Numèric (LaCàN), Department of Mathematics, Polytechnic University of Catalonia, Barcelona, Spain

The paper compares four software packages for the analysis of 3D microscopy images: CellProfiles, Fji/ImageJ, Nikon GA3, and Imaris. The methodology is applied to microwell-based cell culture of pancreatic islets (pseudoislets) and to the analysis with fluorescence microscopy of cell number, cell localisation, and cell-cell interaction with collagen IV fibres.

 Despite the limited biological impact, the conclusions and discussion may be very useful for practitioners that need to analyse microscopy images of 3D aggregates. For instance, the authors conclude that from the three studied microwell arrays  (Aggrwell, Hydrogel, and Elplasia), only the last two showed good single-cell resolution, and that due to strong underestimation of counts, Fiji and CellProfiles where not useful for analysing region detection (core vs mantle).

 The authors give a quantification of quality in terms of cell and nuclei counts and detail the limitations of each software (quality lost along depth or region), and cell separation.

 Yet, the authors need to clarify some unclear points of the manuscript:

- Microscopy Analysis. Manual quantification: authors mention "Fiji cell counter plugin". Is this the same plugin in Fiji, "3D object counter"?
- Statistical Analysis. "The free-to-use statistics program JASP could be used for these analyses." Was this software used? If not please remove the sentence.
- Page 11 of 12, right column: "the cells needed to have <0 μm distance to the collagen IV to be quantified.". Does it mean they had to overlap? If so state this explicitly and clarify.

Is the rationale for developing the new method (or application) clearly explained?

Yes

Is the description of the method technically sound?

Partly

Are the conclusions about the method and its performance adequately supported by the findings presented in the article?

Yes

If any results are presented, are all the source data underlying the results available to ensure full reproducibility?

Partly

Are sufficient details provided to allow replication of the method development and its use by others?

Yes

Reviewer Expertise:

Biomechanics, Cell rheology, Tissue mechanics, Numerical Analysis, Optimisation.

I confirm that I have read this submission and believe that I have an appropriate level of expertise to confirm that it is of an acceptable scientific standard, however I have significant reservations, as outlined above.

# Response

**Published:** 2022-09-13

**Authors:** Timo Rademakers

First of all, we would like to thank the reviewer for their review and the comments provided in order to clarify our methods further. Below, please find our responses to the provided comments:

- Microscopy Analysis. Manual quantification: authors mention "Fiji cell counter plugin". Is this the same plugin in Fiji, "3D object counter"?
  We have clarified in the text that the 2D cell counter, in which cells are manually marked was used, in combination with careful scrolling through the z-stack to prevent double counting of cells. The 3D object counter in Fiji also uses thresholding to detect cells, and in our hands, using the current datasets, was unable to properly segment individual cells. Therefore, a fully manual approach was chosen as a reference.
- Statistical Analysis. "The free-to-use statistics program JASP could be used for these analyses." Was this software used? If not please remove the sentence.
  The program JASP was not used for our analysis. However, it was by request of the journal to add an alternative, free-to-use program to the methods, and according to these guidelines, this sentence was added. We did not change this in the text.
- Page 11 of 12, right column: "the cells needed to have <0 μm distance to the collagen IV to be quantified.". Does it mean they had to overlap? If so state this explicitly and clarify.
  We agree with the reviewer that this indeed required clarification, and altered the text slightly to reflect more clearly the fact that we were interested in interaction of cells with the ECM protein collagen IV. Within the analysis software, this was defined as "<0". As such, we were able to only count cells that indeed were interaction with collagen IV.

# Peer review: Reviewer response for version 1

**DOI:** 10.21956/openreseurope.16096.r29815

**Published:** 2022-09-05

**Authors:** Janne T. Koivisto

**Affiliations:** 1 Division of Pathology, Department of Laboratory Medicine, Karolinska Institutet, Stockholm, Sweden

The manuscript by Fredrik Wieland *et al.* titled "Methodological approaches in aggregate formation and microscopic analysis to assess pseudoislet morphology and cellular interactions" explores the use of four different image analysis software for automated analysis of pancreatic islet cells as 3D organoids. Two of the used software are available for free and two are commercial products. The authors have designed analysis pipelines for each individual software and compared them between each other with manual analysis conducted on Fiji software. The manuscript is clearly written and the topic of this method comparison is highly relevant for the current time on increasing automation of analysis.

 However, I do have a few minor comments for clarifying mainly the Methods chapter:

1. The concentrations of reagents for immunofluorescence staining are in general higher than what is commonly reported, so was the staining protocol of the cell aggregates optimized specifically for this project, or was it based on previously published results?
2.  Why were the fluorescent stainings done with two different methods (inside microwells or collected in microcentrifuge tubes)?
3. How many z-stacks were recorded in total for each case? Amounts of data sets are mentioned in the figure captions in the results but could be also in the Methods chapter.
4. The used software version of Fiji is not mentioned, while other software versions are mentioned.
5. The Discussion chapter could briefly comment more on the user-friendliness of these four compared software and easiness of creating analysis pipelines in them, especially from the point-of-view of a new researcher starting a similar image analysis for the first time and trying to choose the software to be used.

Is the rationale for developing the new method (or application) clearly explained?

Yes

Is the description of the method technically sound?

Yes

Are the conclusions about the method and its performance adequately supported by the findings presented in the article?

Yes

If any results are presented, are all the source data underlying the results available to ensure full reproducibility?

Yes

Are sufficient details provided to allow replication of the method development and its use by others?

Yes

Reviewer Expertise:

Expertise in developing 3D cell culture systems (especially hydrogels), human cell culturing, and 3D imaging

I confirm that I have read this submission and believe that I have an appropriate level of expertise to confirm that it is of an acceptable scientific standard.
